# Differential expression of Notch1 intracellular domain and p21 proteins, and their clinical significance in gastric cancer

**DOI:** 10.3892/ol.2013.1751

**Published:** 2013-12-11

**Authors:** DING-HAI LUO, QIN ZHOU, SUN-KUAN HU, YI-QUN XIA, CHAO-CHAO XU, TIE-SU LIN, YU-TING PAN, JIAN-SHENG WU, RONG JIN

**Affiliations:** 1 Department of Digestive Diseases, The First Affiliated Hospital of Wenzhou Medical College, Wenzhou, Zhejiang 325000, P.R. China; 2 Department of Gastroenterology, Taizhou Hospital, Linhai, Zhejiang 317000, P.R. China; 3 Department of Epidemiology, The First Affiliated Hospital of Wenzhou Medical College, Wenzhou, Zhejiang 325000, P.R. China

**Keywords:** Notch1 intracellular doman, p21, gastric cancer, prognosis, biomarkers

## Abstract

Changes in the expression of the Notch1 intracellular domain (NICD) and p21 proteins have been shown to be closely associated with the development and progression of a number of cancers. The present study aimed to investigate the expression levels of the two proteins in gastric carcinoma and precancerous lesions, and to determine the clinical significance of this. A total of 109 gastric cancer, 57 precancerous gastric lesion, 50 chronic superficial gastritis and 17 normal gastric mucosa patients were recruited for immunohistochemical staining of NICD and p21 protein expression. The protein expression levels in the gastric cancer patient samples were associated with the clinicopathological and survival data. NICD protein levels were upregulated gradually from normal gastric mucosae through chronic superficial gastritis and precancerous gastric lesions to gastric cancer tissues (P<0.01), whereas p21 protein levels were downregulated accordingly (P<0.01). Increased NICD and a loss of p21 expression were closely associated with tumor dedifferentiation, depth of tumor invasion, lymph node metastasis, surface morphology and Lauren classification in gastric cancer. Thus, NICD expression was inversely associated with p21 expression. In addition, the overall survival rate was greater in NICD^−^ and P21^+^ patients than in NICD^+^ and P21^−^ patients, respectively (P<0.05). The COX regression multivariate analysis revealed that NICD^+^, p21^−^, depth of tumor invasion and lymph node metastasis were all independent prognostic factors for patients with gastric cancer. NICD and p21 proteins are differentially expressed in gastric cancer and the aberrant expression of these proteins is associated with an advanced tumor stage, tumor metastasis and overall patient survival. Future studies are required to further evaluate the two proteins as novel prognostic markers for patients with gastric cancer.

## Introduction

Gastric cancer is a significant health problem worldwide, particularly in developing countries, and it accounts for approximately one million new cancer cases per year. In 2008, up to 72% of new cases occurred in developing countries, resulting in 738,000 cancer-related mortalities (1). Furthermore, China alone accounts for 42% of the worldwide gastric cancer cases (2). To date, a number of improvements have been made for early detection and surgical approaches in the treatment of early gastric cancer. The overall five-year survival rate is as high as 95–100% for early cancer patients. However, the majority of patients are diagnosed at an advanced stage of disease, which makes a cure by surgery impossible, leading to a poor overall five-year survival rate. If patients are able to undergo a complete radical surgery, the overall five-year survival rate may reach 30–40%. Therefore, an early diagnosis of gastric cancer and surgery are essential for patients to achieve an improved prognosis. Thus, the development and identification of biomarkers for early detection and prognosis prediction are urgently required.

The Notch1 signaling pathway, an evolutionarily conserved cell interaction mechanism, is involved in embryo development and normal cell proliferation, differentiation, survival and apoptosis, including the induction of radial glia and astrocyte differentiation. However, alterations of this gene pathway contribute to the development of various human cancers and their progression (3,4). Normally, Notch1, a transmembrane protein, is activated by ligand-induced proteolysis, leading to the release of the Notch1 intracellular domain (NICD) from the cytolemma and in turn translocation into the nuclei of cells for controlling the expression of certain genes, including Hes-1 and Hes-5. These downstream target genes are typically regulated through an interaction between NICD and the DNA-binding transcription factor protein, CSL, which maintains normal homeostasis in the human body (5). However, dysregulation of Notch1 or the expression of its functional domain, NICD, may be involved in tumorigenesis. A previous study has shown that abnormal Notch1 signaling contributes to the development and occurrence of gastric cancer (6).

Furthermore, p21/WAF1 protein, also known as cyclin-dependent kinase (CKD) inhibitor 1, is able to bind to and inhibit the activity of cyclin-CDK2 complexes, thus regulating the G1 phase progression of the cell cycle. Normally, p21 expression is tightly controlled by the tumor suppressor protein, p53, to mediate p53-dependent G1 arrest of the cell cycle in response to a variety of stress stimuli. A number of studies have shown that the activation of Notch1 signaling promotes p21 expression in certain types of tumor cells, but inhibits p21 expression in other types (7,15). Specifically, a previous study has shown that Notch signaling induced cell cycle arrest in small cell lung cancer cells (7). Another study has revealed that activated Notch1 interacted with p53 to inhibit its phosphorylation and transactivation (12). In addition, Notch1 has been shown to regulate the Akt signaling pathway and the expression of the cell cycle regulatory proteins cyclin D1, CDK2 and p21 in T-cell acute lymphoblastic leukemia cell lines (15). Therefore, the association between NICD and p21 proteins and the expression of these proteins in gastric cancer development and progression requires further investigation. Thus, in the present study, an immunohistochemical analysis of the two proteins in gastric tissues with varying degrees of histological development was performed to assess their association with gastric cancer.

## Patients and methods

### Tissue specimens

In the present study, 109 surgically resected tissue specimens were retrospectively retrieved from gastric cancer patients who underwent surgery between 2007 and 2009 at The First Affiliated Hospital of Wenzhou Medical College, Wenzhou, China. The patient group comprised 83 males and 26 females, with a mean age of 60.5 years old [standard deviation (SD), ±11.3]. All patients were histopathologically diagnosed with well-differentiated adenocarcinoma (n=5), moderately differentiated adenocarcinoma (n=42) or poorly differentiated adenocarcinoma (n=62) of the stomach. The patients were diagnosed according to the tumor-node-metastasis staging system by the 1997 International Union Against Cancer, with stage I (n=15, 13.8%), stage II (n=18, 16.5%), stage III (n=46, 42.2%) and stage IV (n=30, 27.5%) tumors. No patients were administered any neoadjuvant therapy prior to surgery. In addition, biopsy specimens were obtained from 17 subjects with normal gastric mucosa (who were healthy persons or presented with some symptoms but had histologically normal gastric mucosae), 50 patients with chronic superficial gastritis and 57 patients with precancerous gastric lesions (four cases with a gastric ulcer, two cases with a gastric polyp and 51 cases with chronic atrophic gastritis) through endoscopy. In the normal gastric mucosa group, there were nine males and eight females (mean age ± SD, 42.2±9.8 years). In the chronic superficial gastritis group, there were 27 males and 23 females (mean age ± SD, 40.4±10.4 years) and in the precancerous gastric lesion group (nine chronic atrophic gastritis, 26 atrophic gastritis with intestinal metaplasia, three chronic superficial gastritis with focal areas of atrophic intestinal metaplasia and three atypical hyperplasia patients), there were 35 males and 22 females (mean age ± SD, 47.2±12.4 years). Approval for this study was obtained from the Ethics Review Committee of The First Affiliated Hospital of Wenzhou Medical College. Written informed consent was obtained from each patient. All tissue specimens were fixed in 10% formalin and embedded in paraffin. The patients with gastric cancer were followed up at our outpatient clinic until they succumbed to the disease. The last follow-up appointment was on April 1, 2011.

### Immunohistochemistry

For immunohistochemical staining, the paraffin blocks of each patient were retrieved from the Pathology Department and cut into 3-μm thick sections onto 1% polylysine-coated glass slides. The first section of each block was stained with hematoxylin and eosin to reconfirm the pathological diagnosis. The sections were then stained immunohistochemically using a standard biotin-streptavidin-peroxidase method according to a previous study (16). The primary rabbit anti-human NICD antibody was purchased from Merck-Millipore (Darmstadt, Germany) and diluted at 1:100. The mouse anti-human p21 antibody was obtained from Santa Cruz Biotechnology, Inc. (Santa Cruz, CA, USA) and diluted at 1:50. The secondary antibody and the universal immunohistochemical staining kit (PV6001 and PV9003, respectively) were purchased from Zhongshan Goldenbridge Biotechnology Company (Zhongshan, China).

### Review and scoring of the immunostained tissue sections

The immunostained tissue sections were independently reviewed and scored under a microscope by two pathologists. A brown color or light brown particles in the cytoplasm and/or the nucleus of the cells was considered as positive staining. A total of 10 fields were randomly selected at low magnification (x40) and 100 epithelial cells from each field were counted. The fields were scored as 0 (<1% of the cells stained), one (1–19% staining), two (20–40% staining) or three (>40% staining), according to a previous study (16). p21 protein was reviewed using the same procedure as for NICD, and scored as 0 (<1% of the cells stained), one (1–24% staining), two (25–75% staining) or three (>75% staining), as described previously (16).

### Statistical analyses

All data were analyzed using SPSS 16.0 statistical software (SPSS, Inc., Chicago, IL, USA). The comparison between the groups was analyzed using the χ^2^ test. The correlation of variables was analyzed using the Spearman’s rank correlation test. The survival rates were calculated using the Kaplan-Meier method and compared by the log-rank test. The Cox proportional hazards regression model was used to measure the independent contribution of each variable to the overall survival. P<0.05 was considered to indicate a statistically significant difference.

## Results

### Differential expression of NICD and p21 proteins in gastric cancer, precancerous gastric lesions and normal gastric tissues

**Figure 1:**
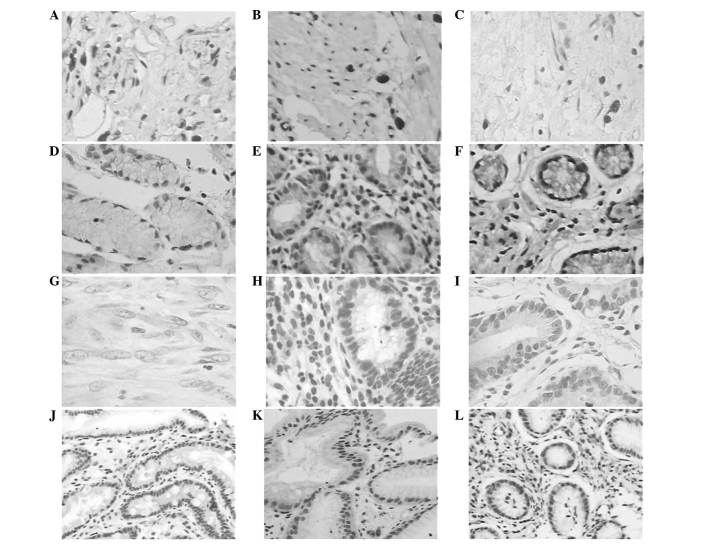
Expression of NICD and p21 proteins in gastric tissues, detected by immunohistochemistry. Expression of NICD protein in (A) poorly differentiated adenocarcinoma; (B) moderately differentiated adenocarcinoma; (C) well-differentiated adenocarcinoma; (D) precancerous gastric conditions, atrophic gastritis with intestinal metaplasia; (E) chronic superficial gastritis; and (F) normal gastric mucosa. Expression of p21 protein in (G) poorly differentiated adenocarcinoma; (H) moderately-differentiated adenocarcinoma,; (I) well-differentiated adenocarcinoma; (J) precancerous gastric conditions, atrophic gastritis with intestinal metaplasia; (K) chronic superficial gastritis; and (L) normal gastric mucosa (magnification, ×400). NICD, Notch1 intracellular domain.

In the present study, NICD expression was first detected in the gastric tissue specimens. The NICD protein was observed to be mainly expressed in the nuclei of epithelial cells and occasionally in the cytoplasm. The NICD protein was expressed in 67.9% (74/109), 36.8% (21/57), 30.0% (15/50) and 23.5% (4/17) of the gastric cancer, precancerous lesion, chronic superficial gastritis and normal gastric mucosa samples, respectively, suggesting that NICD expression was upregulated in the gastric cancer and premalignant lesions. The difference was statistically significant (χ^2^, 30.57; P<0.01). NICD^+^ expression was significantly greater in the gastric cancer samples than in the precancerous lesion (χ^2^, 14.74; P<0.01), chronic superficial gastritis (χ^2^, 19.97; P<0.01) and normal gastric mucosa (χ^2^, 12.27; P<0.01) samples. However, there was no statistically significant difference in NICD expression between the precancerous lesion and chronic superficial gastritis (χ^2^, 0.56; P>0.05) or normal gastric mucosa (χ^2^, 1.04; P>0.05) samples, or between the chronic superficial gastritis and normal gastric mucosa (χ^2^, 0.26; P>0.05) samples. Furthermore, p21 expression was also analyzed in these tissues. The p21 protein was located in the nuclei and was expressed in 38.5% (42/109), 75.4% (43/57), 82.0% (41/50) and 82.4% (14/17) of the gastric cancer, precancerous lesion, chronic superficial gastritis and normal gastric mucosa samples, respectively, suggesting that p21 protein was downregulated from normal mucosae through premalignant lesions to gastric cancer (χ^2^, 40.24; P<0.01). p21^+^ expression was significantly lower in the gastric cancer than in precancerous lesion (χ^2^, 20.40; P<0.01), chronic superficial gastritis (χ^2^, 25.96; P<0.01) and normal gastric mucosa (χ^2^, 11.44; P<0.01) samples. However, there was no significant difference in p21 expression between the precancerous lesion and chronic superficial gastritis samples (χ^2^, 0.68; P>0.05), between the precancerous lesion and normal gastric mucosa samples (χ^2^, 0.35; P>0.05) or between the chronic superficial gastritis and normal gastric mucosa samples (χ^2^, 0.01; P>0.05) (Table I; Fig. 1).

### Association of NICD and p21 expression with clinicopathological features of gastric cancer patients

To assess the clinical significance of NICD and p21 expression, the expression levels of the proteins were analyzed against the clinicopathological features of the gastric cancer patients. The data revealed that NICD protein expression was significantly associated with a larger tumor size (χ^2^, 5.40; P<0.05), tumor dedifferentiation grade (χ^2^, 16.85; P<0.01), depth of tumor invasion (χ^2^, 14.77; P<0.01), lymph node metastasis (χ^2^, 4.82; P<0.05), surface morphology (χ^2^, 13.89; P<0.01) and Lauren classification (χ^2^, 4.60; P<0.05). By contrast, no association with age (χ^2^, 2.45; P>0.05), gender (χ^2^, 1.28; P>0.05), tumor location (χ^2^, 2.53; P>0.05), vascular invasion (χ^2^, 1.13; P>0.05) or distant metastasis (χ^2^, 0.31; P>0.05) was identified. Furthermore, a loss of p21 expression was closely associated with tumor dedifferentiation (χ^2^, 15.45; P<0.01), depth of tumor invasion (χ^2^, 10.75; P<0.01), vascular invasion (χ^2^, 5.12; P<0.05), lymph node metastasis (χ^2^, 5.21; P<0.05), surface morphology (χ^2^, 9.68; P<0.01) and Lauren classification (χ^2^, 7.78; P<0.01). There was also no association with age (χ^2^, 2.20; P>0.05), gender (χ^2^, 0.00; P>0.05), tumor location (χ^2^, 0.80; P>0.05), tumor size (χ^2^, 0.23; P>0.05) or distant metastasis (χ^2^, 0.01; P>0.05) (Table II).

### Association between NICD and p21 expression in gastric cancer

NICD expression was compared with p21 expression in gastric cancer, and the results are provided in Table III. Spearman’s rank correlation test showed that NICD protein expression was inversely associated with p21 protein expression.

### Association of NICD and p21 expression with overall survival of gastric cancer patients

**Figure 2:**
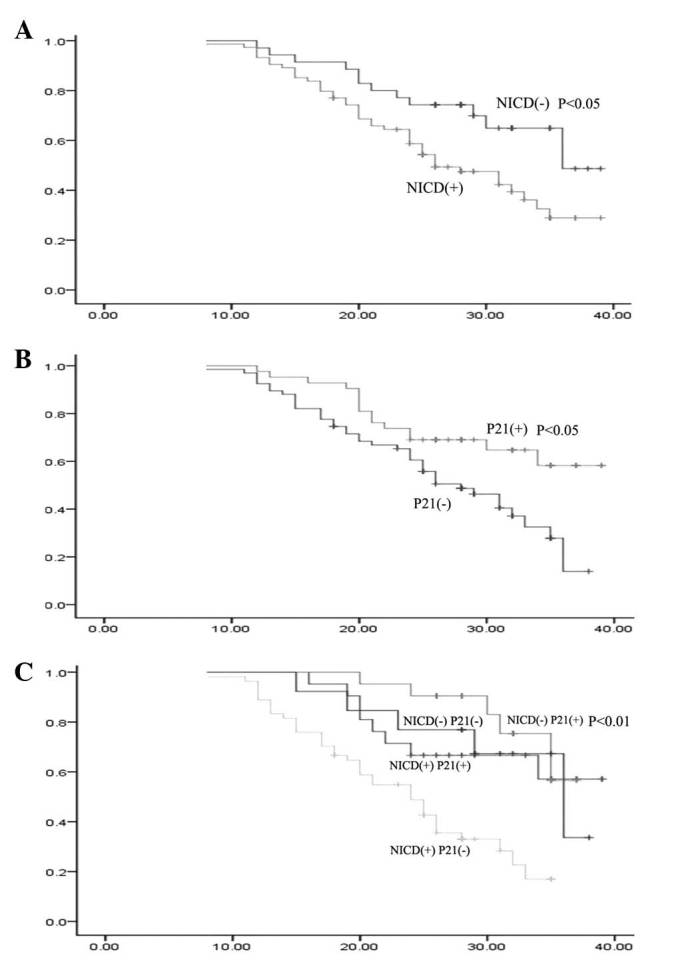
Association of NICD and p21 protein expression with the survival of gastric cancer patients. (A) Overall survival of gastric cancer patients with NICD^+^ or NICD^−^. (B) Overall survival of gastric cancer patients with p21^+^ or p21^−^. (C) Overall survival of gastric cancer patients with NICD^−^/p21^+^, NICD^−^/p21^−^, NICD^+^/p21^+^ or NICD^+^/p21^−^. NICD, Notch1 intracellular domain.

In the present study, gastric cancer patients were followed up for overall survival until April 1, 2011. The overall survival was defined as the time from the surgery to April 1, 2011, provided that the patient survived until that date, or the date of mortality. The 109 gastric cancer patients were followed up for 5–40 months with a mean follow-up time of 21.09±6.82 months, among which there were 55 patients who succumbed to the disease prior to the last follow-up. The two- and three-year survival rates of NICD^+^ (58.7 and 28.9%, respectively) gastric cancer patients were significantly lower than those that were NICD^−^ (74.30 and 48.70%, respectively; χ^2^, 6.01; P<0.05; Fig. 2A). The three-year survival rate of gastric cancer patients with p21^+^ (58.3%) expression was significantly greater than that of p21^−^ patients (13.90%) (χ^2^, 6.84; P<0.05; Fig. 2B). The survival rates were determined using the expression data of NICD and p21, and the two- and three-year survival rates were 90.5 and 57.1%, 67.0 and 33.7%, 66.7 and 56.5% and 48.8 and 17.0% in NICD^−^/p21^+^, NICD^−^/p21^−^, NICD^+^/p21^+^ and NICD^+^/p21^−^ patients, respectively. Furthermore, the survival rate in NICD^−^/p21^+^ patients was significantly higher than that of NICD^+^/p21^−^ patients (χ^2^, 15.57; P<0.01; Fig. 2C).

### Univariate and multivariate analyses of prognostic factors for overall survival of gastric cancer patients

Univariate and multivariate analyses of prognostic factors were performed for overall survival of gastric cancer patients using the Cox proportional hazards regression model. Among the 11 factors that were analyzed (age, gender, tumor location, tumor size, tumor differentiation, depth of tumor invasion, vascular invasion, lymph node and distant metastasis, and NICD and p21 protein expression; Table IV), the univariate analysis showed that tumor differentiation, depth of tumor invasion, vascular invasion, lymph node metastasis, and NICD^+^ and p21^+^ protein expression were eligible for the multivariate analysis (Table V). The multivariate analysis revealed that only NICD^+^ or p21^+^ protein expression, depth of tumor invasion and lymph node metastasis had statistical significance. NICD^+^ or p21^−^ protein expression, depth of tumor invasion and lymph node metastasis were independent prognostic factors of gastric cancer (Tables III and IV).

## Discussion

The present study identified differential expression of the NICD and p21 proteins in gastric cancer tissue specimens compared with in normal mucosa, gastritis and precancerous lesion samples. NICD was upregulated, but p21 protein was downregulated, in the gastric cancer tissues, and the two proteins were shown to be inversely associated. Furthermore, increased NICD expression, but a loss of p21 expression, was closely associated with tumor dedifferentiation, depth of tumor invasion, lymph node metastasis, surface morphology and Lauren classification in gastric cancer. The overall survival rate of gastric cancer patients was greater in those with NICD^−^ as opposed to NICD^+^ tumors, and in p21^+^ rather than in p21^−^ tumors. The altered expression of these two proteins was also associated with the overall survival of the patients. The COX-regression multivariate analysis showed that NICD^+^, p21^−^, depth of tumor invasion and lymph node metastasis were all independent prognostic factors for gastric cancer patients. Future studies will further evaluate these two proteins as novel prognostic markers for gastric cancer patients.

Using a pancreatic cancer mouse model (Rosa^26NICD^), De La *et al*(18) demonstrated that the abnormal activation of Notch1 signaling leads to excessive epithelial cell proliferation, decreased apoptosis and malignant transformation of the epithelial phenotype, consequently resulting in the development of pancreatic intraepithelial neoplasms and cancer in the mice. The present study showed that NICD protein expression was significantly greater in poorly-differentiated gastric cancer compared with that in well- and moderately differentiated tumors. Furthermore, NICD expression was closely associated with tumor size, depth of tumor invasion, lymph node metastasis, surface morphology and Lauren classification of tumors. These *ex vivo* data are consistent with the previously mentioned data on pancreatic cancer in mice. Similarly, Fre *et al*(19) identified that the overexpression of NICD through transgenic technology significantly inhibited the differentiation of crypt progenitor cells in the mouse intestine. In glioma, Fan *et al*(20) demonstrated that the inhibition of Notch1 signaling activation reduced the proportion of glioma stem cells, inhibited tumor cell colony formation and increased tumor cell differentiation and apoptosis. In gastric cancer, Yeh *et al*(21) revealed that the overexpression of the NICD protein in gastric adenocarcinoma SC-M1 cells using gene transfection techniques resulted in a marked increase in tumor cell colony formation, migration, invasion, xenograft formation and growth. Recently, Notch1 protein expression has been shown to regulate stem cells and cancer stem cells. The constitutive activation of Notch1 signaling in Sertoli cells has been shown to cause gonocytes to exit from quiescence (22). Notch overexpression has been demonstrated to preserve stem cell characteristics and confer stem cell characteristics upon a subset of progenitor cells (23). Furthermore, Notch1 is able to promote T cell leukemia-initiating activity by RUNX-mediated regulation of PKC-θ and reactive oxygen species (24). However, Notch1 inhibition *in vivo* results in mammary tumor regression and reduces mammary tumor sphere-forming activity *in vitro*(25). The inhibition of the Notch1 pathway has been shown to allow glioblastoma cells to overcome apoptosis resistance and become sensitized to apoptosis that is induced by ionizing radiation, the death ligand tumor necrosis factor-related apoptosis-inducing ligand or the Bcl-2/Bcl-XL inhibitor ABT-737 (26). In conclusion, Notch1 may be a novel target for gastric cancer therapy.

Furthermore, p21 expression has been shown to be reduced or lost in a variety of cancer types (27,28,29). A possible explanation is that p21 functions as a regulator of cell cycle progression at S phase, therefore preventing cell proliferation. In addition, p21 expression is controlled by the tumor suppressor protein, p53, which is frequently mutated in a number of human cancers, thus significantly contributing to a loss of p21 expression in various cancer tissues. In the present study, a gradual reduction of p21 protein expression from normal gastric mucosa, chronic superficial gastritis and precancerous gastric lesions to gastric cancer was observed. The loss of p21 expression was associated with tumor dedifferentiation, depth of tumor invasion, vascular invasion, lymph node metastasis, surface morphology and Lauren classification of gastric cancer. These data suggest that p21 plays a suppressor role in the development and progression of gastric cancer, the expression of which may aid in controlling a variety of malignant behaviors of gastric cancer. Furthermore, the effect of activated Notch1 signaling (NICD) on the regulation of p21 expression may differ in various tumor cell types. However, the majority of studies support that Notch1 expression inhibits p21 expression and activation or vice versa (30). p21WAF1/Cip1 is a negative transcriptional regulator of Wnt4 expression downstream of Notch1 activation (31). The adult stem cell marker Musashi-1 modulates endometrial carcinoma cell cycle progression and apoptosis via Notch1 and p21 (32). Silencing of SKP2 by RNA interference in G1 stabilizes p27 and p21 but abolishes the Notch1 effect on G1-S progression (33). Kim *et al*(12) also observed that the overexpression of NICD inhibits p53 phosphorylation and the expression of the p53 target gene, p21, therefore inhibiting ultraviolet-induced apoptosis. These data indicate that Notch1 may function by regulating p21 expression. The present study supports this notion. However, further studies are required to clarify Notch regulation of p21 expression in gastric cancer cells.

The present study demonstrated that a combination of aberrant expression of NICD and p21 proteins was able to predict overall survival of gastric cancer patients, which is more efficient than that of an individual protein. The present data are consistent with the data reported by Li *et al*(34). Thus, the NICD and p21 proteins may be useful as prognostic indicators for gastric cancer. However, the present data showed that the expression of the two proteins was significantly altered in gastric cancer tissues, although they were not significantly altered in the early stages of malignancy, including precancerous lesions versus chronic superficial gastritis or normal gastric mucosae, or chronic superficial gastritis versus normal gastric mucosae, indicating that they may be late events during stomach carcinogenesis. Thus, they are not useful for early detection or as tumorigenesis markers of gastric cancer.

## Figures and Tables

**Table I:** Differential expression of NICD and p21 proteins in gastric tissue specimens.

A, Positive rate of NICD and p21 proteins in differential specimens.							

Group	n	NICD^+^, n (%)	χ^2^	P-value	p21^+^, n (%)	χ^2^	P-value
Gastric cancer	109	74 (67.89)	30.57	P<0.01	42 (38.53)	40.24	P<0.01
Precancerous lesions	57	21 (36.84)			43 (75.44)		
Chronic superficial gastritis	50	15 (30.00)			41 (82.00)		
Normal gastric mucosa	17	4 (23.53)			14 (82.35)		

B, Comparison between differential groups using χ^2^ test.				

Comparison	χ^2^	P-value	χ^2^	P-value
Cancer vs. precancerous lesions	14.47	<0.01	20.40	<0.01
Cancer vs. chronic superficial gastritis	19.97	<0.01	25.96	<0.01
Cancer vs. normal gastric mucosa	12.27	<0.01	11.44	<0.01
Precancerous lesions vs. chronic superficial gastritis	0.56	>0.05	0.68	>0.05
Precancerous lesions vs. normal gastric mucosa	1.04	>0.05	0.35	>0.05
Chronic superficial gastritis vs. normal gastric mucosa	0.26	>0.05	0.01	>0.05

NICD, Notch1 intracellular domain.

**Table II:** Association of NICD and p21 expression with the clinicopathological features of gastric cancer patients.

Group	n	NICD^+^, n (%)	χ^2^	P-value	p21^+^, n (%)	χ^2^	P-value
Age (years)							
<60	46	35 (76.9)	2.45	>0.05	14 (30.4)	2.20	>0.05
≥60	63	39 (61.9)			28 (44.4)		
Gender							
Male	83	54 (65.1)	1.28	>0.05	32 (38.6)	0.00	>0.05
Female	26	20 (76.9)			10 (38.5)		
Location							
Cardia and fundus	18	12 (66.7)	2.53	>0.05	7 (38.9)	0.80	>0.05
Gastric body	39	29 (74.4)			14 (35.9)		
Angular region	12	6 (50.0)			6 (50.0)		
Antrum and pylorus	40	27 (67.5)			15 (37.5)		
Tumor size (cm)							
<3	42	23 (54.8)	5.40	<0.05	15 (35.7)	0.23	>0.05
≥3	67	51 (76.1)			27 (40.3)		
Differentiation							
Well and moderate	47	22 (46.8)	16.85	<0.01	28 (59.6)	15.45	<0.01
Poor	62	52 (83.9)			14 (22.6)		
Depth of tumor invasion							
T1+T2	30	20 (66.7)	14.77	<0.01	19 (63.3)	10.75	<0.01
T3+T4	79	55 (69.6)			23 (29.1)		
Vascular invasion							
Positive	64	46 (71.9)	1.13	>0.05	19 (29.7)	5.12	<0.05
Negative	45	28 (62.2)			23 (51.1)		
Lymph node metastasis							
Positive	69	52 (75.4)	4.82	<0.05	21 (30.4)	5.21	<0.05
Negative	40	22 (55.0)			21 (52.5)		
Distant metastasis							
Positive	10	6 (60.0)	0.31	>0.05	4 (40.0)	0.01	>0.05
Negative	99	68 (68.7)			38 (38.3)		
Surface morphology							
Early gastric cancer	11	2 (18.2)	13.89	<0.01	9 (81.8)	9.68	<0.01
Progressive gastric cancer	98	72 (73.5)			33 (33.7)		
Lauren Classification							
Intestinal type	65	39 (60.0)	4.60	<0.05	32 (49.2)	7.78	<0.01
Diffused type	44	35 (79.6)			10 (22.7)		

NICD, Notch1 intracellular domain.

**Table III:** Association of NICD and p21 protein expression.

Group	n (%)	χ^2^	P-value
NICD^+^/p21^+^	20 (18.35)	7.40	P<0.01
NICD^−^/p21^−^	13 (11.93)		
NICD^+^/p21^−^	54 (49.54)		
NICD^−^/p21^+^	22 (20.18)		

NICD, Notch1 intracellular domain.

**Table IV:** Univariate analysis of prognostic factors for the overall survival of gastric cancer patients.

						95% confidence bounds	
						
Variable	β	SE	Wald	P-value	OR	Lower limit	Upper limit
Age (years)	−0.15	0.28	0.29	0.59	0.86	0.50	1.47
Gender (n)	0.12	0.32	0.13	0.71	1.12	0.60	2.09
Tumor size (cm)	−0.34	0.30	1.29	0.26	0.71	0.40	1.28
Tumor location (n)	−0.01	0.13	0.00	0.96	0.99	0.77	1.29
Differentiation (n)	0.60	0.29	4.36	0.04	1.83	1.04	3.22
Depth of tumor invasion (n)	1.34	0.29	21.34	0.00	3.80	2.16	6.71
Vascular invasion (n)	0.71	0.30	5.71	0.02	2.04	1.14	3.65
Lymph node metastasis (n)	1.04	0.33	9.74	0.00	2.82	1.47	5.41
Distant metastasis (n)	0.45	0.41	1.22	0.27	1.57	0.71	3.47
NICD protein (n)	1.19	0.35	11.34	0.00	3.27	1.64	6.53
p21 protein (n)	−1.14	0.32	12.58	0.00	0.32	0.17	0.60

β, coefficient of regression; SE, standard error of the mean; OR, odds ratio; NICD, Notch1 intracellular domain.

**Table V:** Multivariate analysis of prognostic factors for the overall survival of gastric cancer patients.

						95% confidence bounds	
						
Variable	β	SE	Wald	P-value	OR	Lower limit	Upper limit
Tumor differentiation	0.10	0.31	0.11	0.74	1.11	0.60	2.03
Depth of tumor invasion	1.10	0.30	14.29	0.00	3.00	1.70	5.31
Vascular invasion	−0.10	0.32	0.10	0.76	0.91	0.49	1.69
Lymph node metastasis	1.66	0.41	16.12	0.00	5.23	2.33	11.73
NICD protein	0.83	0.39	4.39	0.04	2.28	1.06	4.94
p21 protein	−0.70	0.34	4.37	0.04	0.50	0.26	0.96

β, coefficient of regression; SE, standard error of the mean; OR, odds ratio; NICD, Notch1 intracellular domain.
